# Low density lipoprotein receptor related protein 1 regulates adult hippocampal neurogenesis and memory

**DOI:** 10.1002/alz.094784

**Published:** 2025-01-09

**Authors:** Kristi Dietert, Nicole Marion, Erzsebet Kokovay, Naomi L. Sayre

**Affiliations:** ^1^ University of Texas Health San Antonio, San Antonio, TX USA

## Abstract

**Background:**

Adult neural stem cells are fundamental in allowing the brain to retain memory via hippocampal neurogenesis. In Alzheimer’s disease, patients have impaired neurogenesis, which may contribute to memory impairments. We have discovered a new player in the brain’s ability to retain memory: the protein LRP1 (**low density lipoprotein receptor related protein**). LRP1 is a promiscuous membrane receptor which differentially regulates cell signaling through multiple mechanisms. LRP1 is implicated in Alzheimer’s disease pathogenesis via its interaction with disease related proteins, including ApoE4, tau, and amyloid beta.

**Methods:**

We tested a role for LRP1 in adult hippocampal neurogenesis *in vivo* via Cre‐mediated recombination to knockout LRP1 from adult neural stem cells. We generated mice which expressed a tamoxifen‐inducible Cre under control of the Nestin promotor which were crossed with mice with stop‐floxed inducible td‐tomato and floxed LRP1 (Nestin‐Cre^ERt2^:td‐tomato^fl/fl^:LRP1^fl/fl^). Adult (3‐month‐old) mice were subjected to tamoxifen treatment to induce Cre expression and so cause expression of td‐tomato and knockout of LRP1 in neural stem cells to generate **LRP1KO** mice. As a control, we used mice one generation removed which lacked floxed LRP1 (Nestin‐Cre^ERt2^:td‐tomato^fl/fl^:LRP1^+/+^). Mice (n = 10‐20 males and females) were aged to either 4‐ or 9‐months, and cognitive impairment was measured via a battery of tests including the elevated plus maze, Y maze, and the Barnes maze. Tissues were harvested for subsequent analysis.

**Results:**

LRP1KO mice displayed significant impairments in cognition compared to control mice, including increased anxiety resulting in reduced open arm time in the elevated plus maze (p = 0.02), reduced spontaneous alterations in the Y‐maze (p = 0.01), and reduced target exploration in the Barnes maze (p = 0.02). Tissue analysis revealed that while neural stem cell proliferation was consistent between LRP1KO and control mice, greater numbers of td‐tomato positive, NeuN positive mature neurons were observed in 9‐month old mice (p<0.001), suggesting increased differentiation of these cells into neurons.

**Conclusion:**

LRP1 regulates adult hippocampal neurogenesis in a manner which is protective of memory, and impairments to LRP1 function disrupts hippocampal neurogenesis. These findings may have significant relevance towards Alzheimer’s disease pathogenesis, as impairments to LRP1 function can occur in the context of ApoE4/amyloid beta/tau expression and in aging.

